# Correction: Sato et al. Separation of Fructosyl Oligosaccharides in Maple Syrup by Using Charged Aerosol Detection. *Foods* 2021, *10*, 3160

**DOI:** 10.3390/foods12112220

**Published:** 2023-05-31

**Authors:** Kanta Sato, Tetsushi Yamamoto, Kuniko Mitamura, Atsushi Taga

**Affiliations:** Pathological and Biomolecule Analyses Laboratory, Faculty of Pharmacy, Kindai University, 3-4-1 Kowakae, Higashiosaka 577-8502, Japan

## Error in Figure 1

In the original publication [1], there was a mistake in Figure 1 as published. Fructose residues on 1-kestose, nystose, and 1-fructofuranosylnystose lack methylene groups, and these structures are incorrectly drawn sterically. The corrected Figure 1. The structure of 1-kestose, nystose, and 1-fructofuranosylnystose as the representative FOS. appears below. 

## Error in Table 3

In the original publication, there was a mistake in Table 3. as published. The proton signals of C-6 in glucose and C-1, -6 in fructose residue, as a constitute of neokestose, are missing and incomplete. The corrected Table 3. ^1^H (800 MHz) and ^13^C (200 MHz)-NMR spectral data of neokestose in D_2_O. appears below. 

## Error in Figure 4

In the original publication, there was a mistake in Figure 4. as published. Fructose residues of neokestose are drawn as L-fructose. The corrected Figure 4. The structure of neokestose. appears below. 

## Reference Correction

In the original publication, reference [2] and [3] was not cited. The citation has now been inserted in “[28]” and “[30]”.

## Text Correction

There was an error in the original publication. Since neokestose had been found in maple sap, we believe that the word “identified” is more appropriate than “discovered”.

A correction has been made to “**Abstract**”:

“Fructosyl oligosaccharides, including fructo-oligosaccharide (FOS), are gaining popularity as functional oligosaccharides and have been found in various natural products. Our previous study suggested that maple syrup contains an unidentified fructosyl oligosaccharide. Because these saccharides cannot be detected with high sensitivity using derivatization methods, they must be detected directly. As a result, an analytical method based on charged aerosol detection (CAD) that can detect saccharides directly was optimized in order to avoid relying on these structures and physical properties to clarify the profile of fructosyl oligosaccharides in maple syrup. This analytical method is simple and can analyze up to hepta-saccharides in 30 min. This analytical method was also reliable and reproducible with high validation values. It was used to determine the content of saccharides in maple syrup, which revealed that it contained not only fructose, glucose, and sucrose but also FOS such as 1-kestose and nystose. Furthermore, we identified neokestose as a maple syrup content. It has only been found in a few natural foods as a fructosyl oligosaccharide. These findings help to shed light on the saccharides profile of maple syrup.”

There was an error and “[2]” was not cited in the original publication. The citation has now been inserted in “[28]”. Since neokestose had been found in maple sap, we believe that the word “identified” is more appropriate than “discovered”. In addition, we would like to cite a previous report by Haq, S., et al.

A correction has been made to “**Introduction**”, Paragraph 4:

“This study aims to optimize the analysis method using FOS standards by hydrophilic interaction chromatography (HILIC)-CAD to clarify the profile of fructosyl oligosaccharides, including FOS in maple syrup. This optimized method is simple, compact, reliable, and reproducible. While the RID method could only detect sucrose, glucose, and fructose, this optimized method using CAD was able to observe the several rare saccharides as well as the major saccharides such as sucrose. In addition, its quick application was used to identify and quantify several FOS in maple syrup precisely. Furthermore, we showed that maple syrup contained neokestose [28], which is rarely reported in natural foods, as a constituent of maple syrup.”

There was an error in the original publication. The word “mapletriose” is a pseudonym within this manuscript and may confuse the reader of this manuscript if it is not clearly stated.

A correction has been made to “*3.3. Analysis of Carbohydrates Observed in Maple Syrup*”, Paragraph 2:

“On the other hand, any unidentified peaks were observed at 18–21 min of this chromatogram, which was predicted to be a trisaccharide based on the retention time. We called these unidentified saccharides “mapletrioses” in this manuscript. Mapletrioses were identified as three distinct peaks and were numbered in ascending order of retention time. Mapletriose1 was the fourth highest peak in maple syrup after sucrose, glucose, and fructose, and it slightly overlapped with the peak of 1-kestose. Mapletriose1 and 1-kestose had a resolution of 1.4. On the other hand, mapletriose2 and 3 were present in trace amounts. Although we attempted to identify mapletrioses using standards available on the general reagent market, these saccharides were unable to be identified.”

There was an error and [3] was not cited in the original publication. The citation has now been inserted in “[30]”. We believe that the statement regarding LC-ESI-MS/MS analysis needs to be modified because it needs to be stated in the Structural Analysis section, not in the Discussion section. In addition, the text needs to be changed to reflect the correction in Table 3. 

A correction has been made to “*3.4. Structural Analysis of Mapletriose1 by NMR”, Paragraph 1:*

“The LC-ESI-MS/MS spectrum of mapletriose1, which was isolated from maple sap according to the method reported by us [12], showed a peak at *m*/*z* 503.1 due to a quasi-molecular ion [M–H]^–^, suggesting that mapletriose1 has a trisaccharide structure. Its 200 MHz ^13^C NMR spectrum showed 18 peaks reasonably related to the 18 carbons of a trisaccharide as shown in Table 3. Chemical shift values of these ^13^C NMR signals showed perfect correlations with those of β-D-fructofuranosyl 6-*O*-β-D-fructofuranosyl-β-D-glucopyranoside (neokestose) [30] with respect to all the signals with deviations within *ca*. 0.1 ppm. The connections from C1-F to C6-F and from C1”-F to C6”-F of two D-fructofuranose units as well as that from C1’-G to C6’-G of D-glucopyranose unit were clearly confirmed by COSY, HSQC, and HMBC experiments. All signals in the 800 MHz ^1^H NMR spectrum were also unambiguously assigned as shown in Table 3. However, signals due to C2-F and C2″-F carbons were indistinguishable from each other because of their very close chemical shift values (*δ*_C_ 106.42 and *δ*_C_ 106.43). Therefore, although the signals due to C2-F and C2″-F carbons showed HMBC correlations with signals due to H1-G (*δ*_H_ 5.39) and H6-G (*δ*_H_ 3.92) protons of D-glucose, the detailed assignment regarding the connecting positions of the fructose units with the D-glucose unit was impossible. Fortunately, the depicted structure of neokestose in Figure 4 was supported by the NOESY correlations between H4-F (*δ*_H_ 4.05) and H1-G protons.”

There was an error and [2] was not cited in the original publication. The citation has now been inserted in “[28]”. We believe that it is necessary to remove some sentences because of the duplication of content regarding the structural analysis of the neokestose. In addition, we would like to cite a previous report by Haq, S., et al.

A correction has been made to “**Discussion**”, Paragraph 3.

“Our previous study found blastose in maple syrup [12]. This study also showed that blastose levels were rising due to invertase digestion of maple syrup. Invertase, also known as β-fructofuranosidase, can cleave a glycosyl bond between fructose and glucose from the fructose side. This occurrence suggested that maple syrup contained an unidentified fructosyl oligosaccharide that was considered to be fructosyl blastose. On the other hand, blastose is a disaccharide composed of fructose and glucose with a β(2→6) linkage, and its structure is not shared by 1-kestose or nystose, but neokestose, which is a fructosylated blastose having β(2→6) linkage, is also known as neo-inulin type fructosyl oligosaccharide or neo-FOS. Although neokestose was isolated from maple sap by Haq, S., et al., in 1961 [28] and was reported as a transfructosylation product by levansucrase [34,35], few papers have reported on the qualitative and quantitative analyses of neokestose in natural foods, because its standard is not available on the general reagent market.”

There was an error in the original publication. Since neokestose is a known compound, we believe that the word “identified” is more appropriate than “discovered”. 

A correction has been made to “**Conclusions**”, Paragraph 1.

“In this study, we used CAD to optimize the analysis methods for fructosyl oligosaccharides in order to clarify the saccharide profile in maple syrup. This method is simple and compact, and it can analyze up to hepta-saccharides within 30 min. In addition, we used this analytical method to precisely determine the mono- and di-saccharides and fructosyl oligosaccharides, including FOS, in maple syrup of all grades. Maple syrup contained sucrose, glucose, and fructose as major saccharides; it also contained FOS such as 1-kestose and nystose. Furthermore, we identified a fructosyl oligosaccharide neokestose in maple syrup. This fructosyl oligosaccharide is rarely reported in natural foods. Because of their structural characteristics, these fructosyl oligosaccharides, including FOSs, may be related to the function of maple syrup.”

The authors apologize for any inconvenience caused and state that the scientific conclusions are unaffected. The original publication has also been updated.

## Figures and Tables

**Figure 1:**
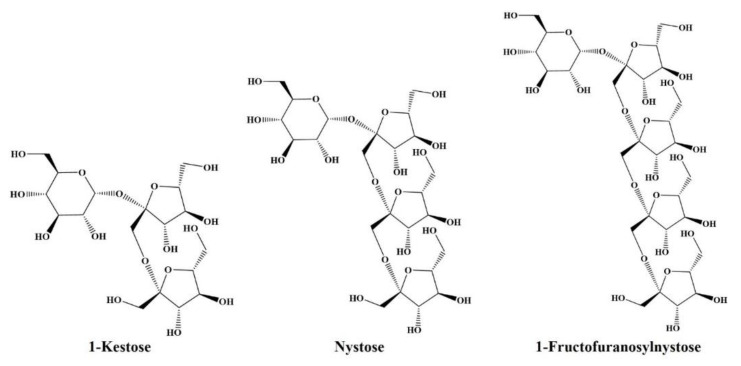
The structures of 1-kestose, nystose, and 1-fructofuranosylnystose as the representative FOS.

**Figure 4:**
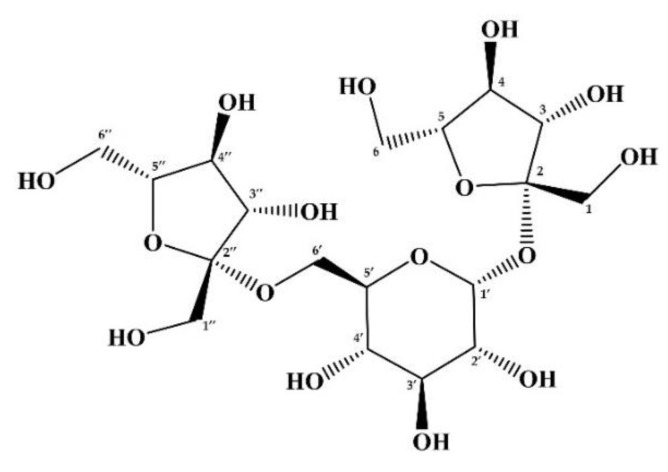
The structure of neokestose.

**Table 3:** ^1^H (800 MHz) and ^13^C (200 MHz)-NMR spectral data of neokestose in D_2_O.

Chemical Shift			
Residue	Position	Observed (200 MHz) in D_2_O *δ*_C_	Observed (800 MHz) in D_2_O *δ*_H_
Fruf β	1	64.15	3.64 (d, 12.6)
			3.656 (d, 12.6)
	2	106.42 *	-
	3	78.92	4.21 (d, 8.9)
	4	76.64	4.05 (dd-like, ca. 8.9, ca. 8.5)
	5	84.07	3.88 (ddd, 8.5, 7.3, 3.0)
	6	65.14	3.78 (dd-like, ca. 12.4, ca. 7.3)
			3.83 (dd-like, ca. 12.4, 3.0)
Glcp α	1′	94.72	5.39 (d, 3.9)
	2′	73.73	3.55 (dd, 9.9, 3.9)
	3′	75.15	3.736 (dd-like, ca. 9.9, ca. 9.5)
	4′	71.89	3.51 (dd-like, ca. 10.3, ca. 9.5)
	5′	74.25	3.93 (ddd-like, ca. 10.3, 4.1, 2.1)
	6′	63.02	3.78 (dd-like, ca. 11.5, ca. 4.1)
			3.92 (dd-like, ca. 11.5, 2.1)
Fruf β	1″	62.90	3.660 (d, 12.1)
			3.744 (d, 12.1)
	2″	106.43 *	-
	3″	79.48	4.18 (d, 8.7)
	4″	77.04	4.13 (dd-like, ca. 8.7, ca. 7.8)
	5″	83.86	3.86 (ddd, 7.8, 6.6, 3.0)
	6″	65.07	3.69 (dd, 12.4, 6.6)
			3.82 (dd-like, ca. 12.4, 3.0)

* signals are not determined.
