# Supplementary material for: Separation of Fructosyl Oligosaccharides in Maple Syrup by Using Charged Aerosol Detection
Source: Foods. 2021 Dec 20;10(12):3160. doi: 10.3390/foods10123160 (PMC8701490; doi:10.3390/foods10123160)
Supplement: Supplementary file 1 [file foods-10-03160-s001.zip › foods-1472883-supplementary.pptx]

## Slide 1
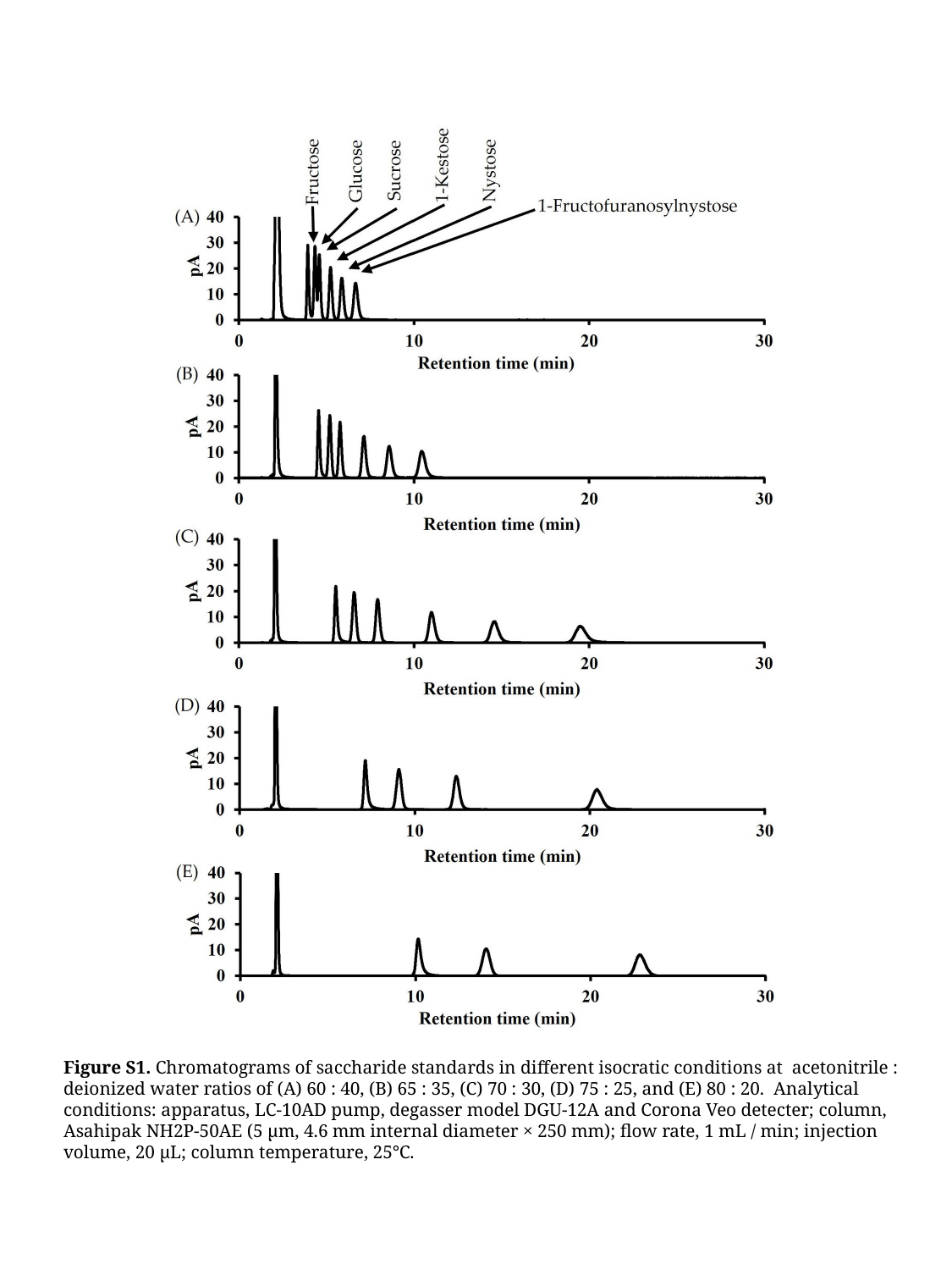

Figure S1. Chromatograms of saccharide standards in different isocratic conditions at acetonitrile : deionized water ratios of (A) 60 : 40, (B) 65 : 35, (C) 70 : 30, (D) 75 : 25, and (E) 80 : 20. Analytical conditions: apparatus, LC-10AD pump, degasser model DGU-12A and Corona Veo detecter; column, Asahipak NH2P-50AE (5 μm, 4.6 mm internal diameter × 250 mm); flow rate, 1 mL / min; injection volume, 20 μL; column temperature, 25℃.

## Slide 2
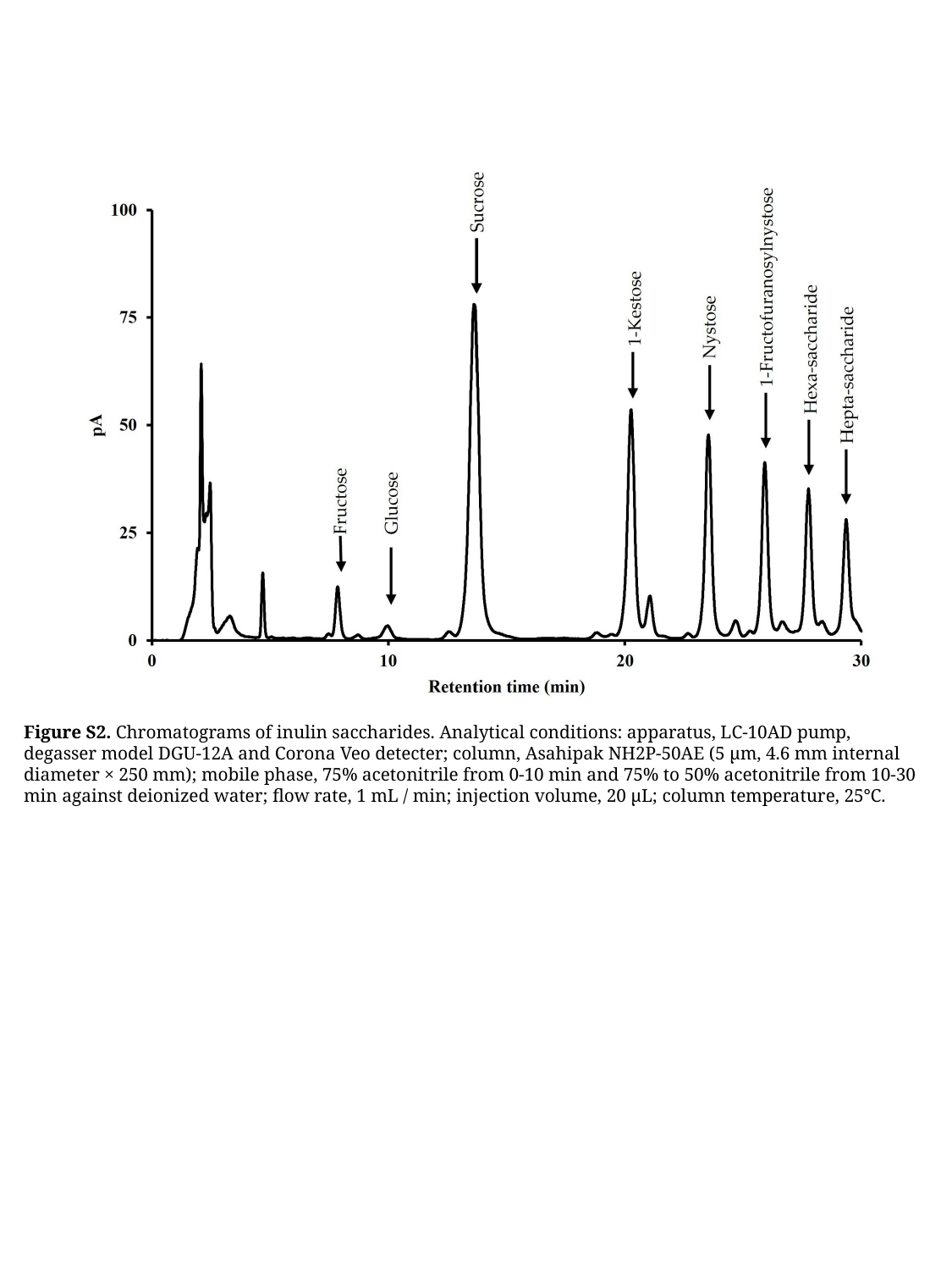

Figure S2. Chromatograms of inulin saccharides. Analytical conditions: apparatus, LC-10AD pump, degasser model DGU-12A and Corona Veo detecter; column, Asahipak NH2P-50AE (5 μm, 4.6 mm internal diameter × 250 mm); mobile phase, 75% acetonitrile from 0-10 min and 75% to 50% acetonitrile from 10-30 min against deionized water; flow rate, 1 mL / min; injection volume, 20 μL; column temperature, 25℃.

## Slide 3
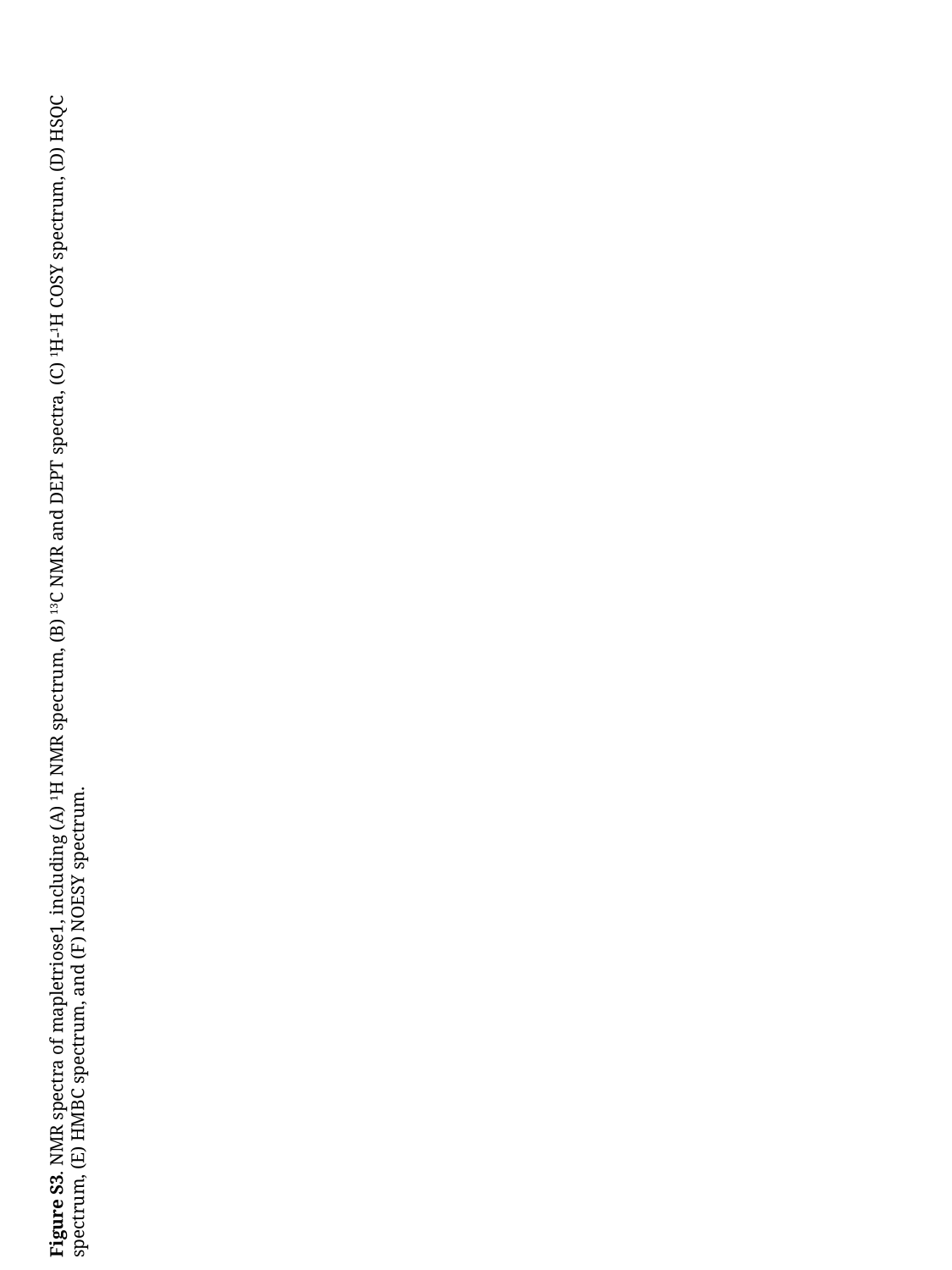

Figure S3. NMR spectra of mapletriose1, including (A) 1H NMR spectrum, (B) 13C NMR and DEPT spectra, (C) 1H-1H COSY spectrum, (D) HSQC spectrum, (E) HMBC spectrum, and (F) NOESY spectrum.

## Slide 4
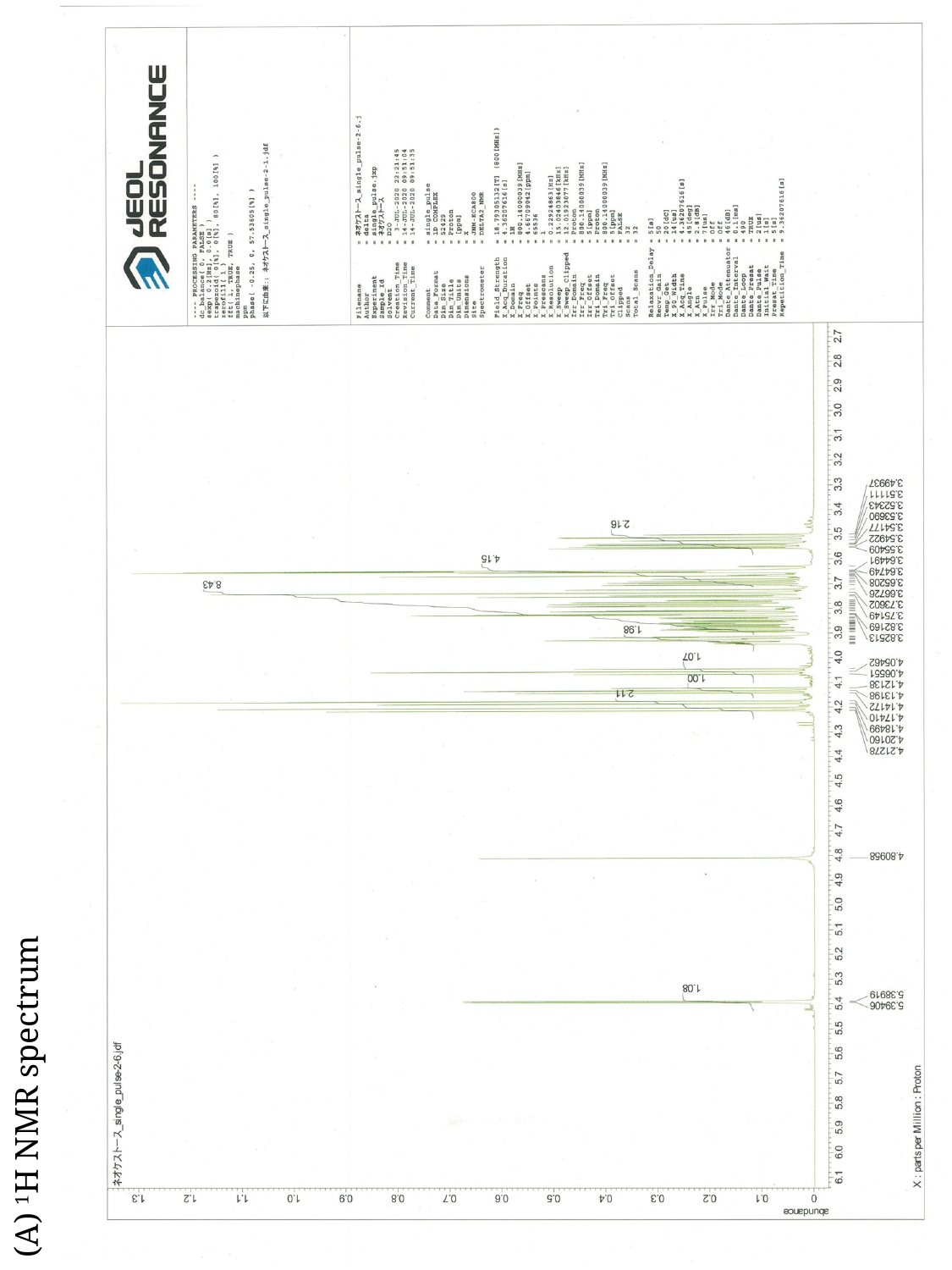

(A) 1H NMR spectrum

## Slide 5
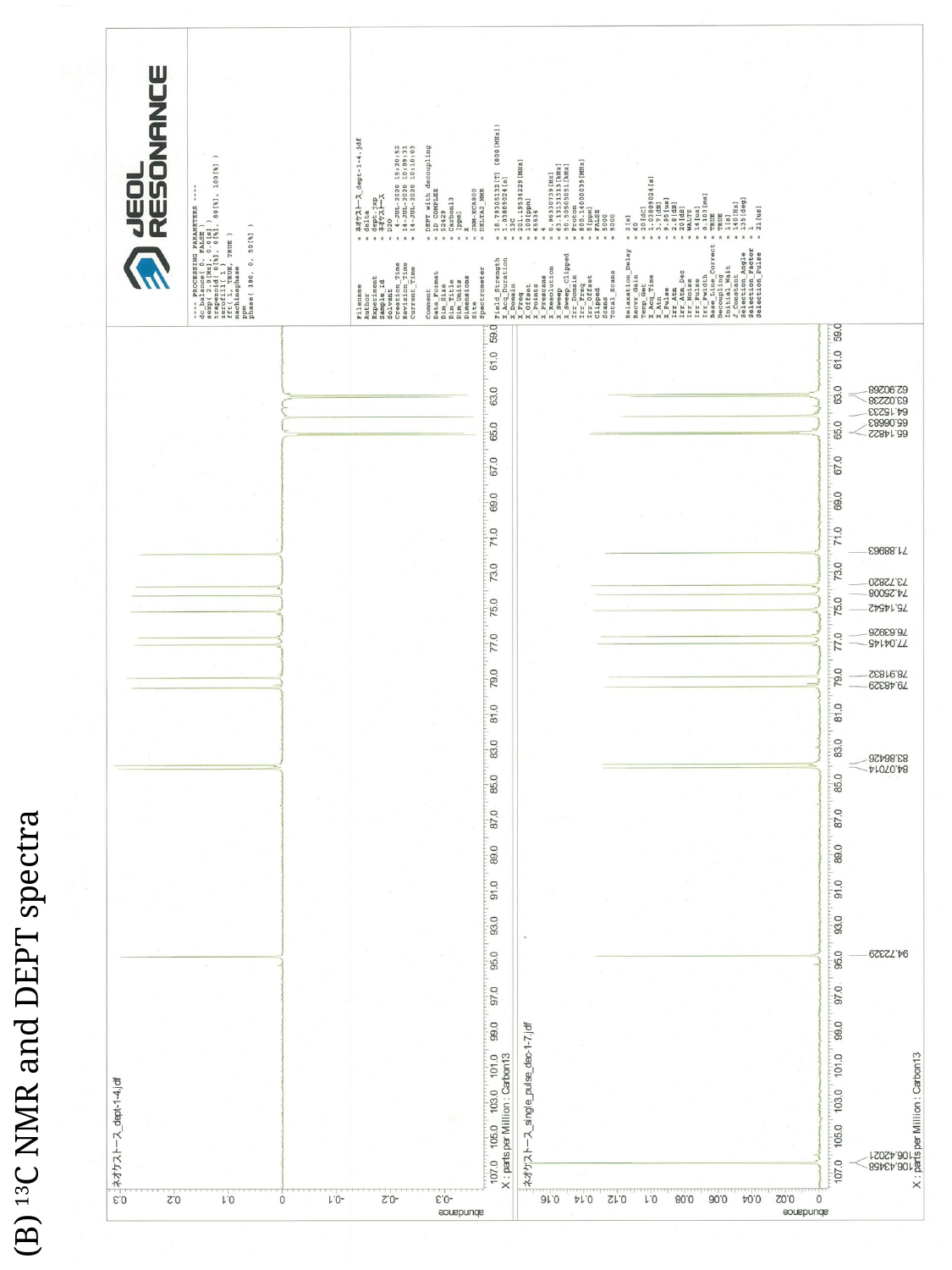

(B) 13C NMR and DEPT spectra

## Slide 6
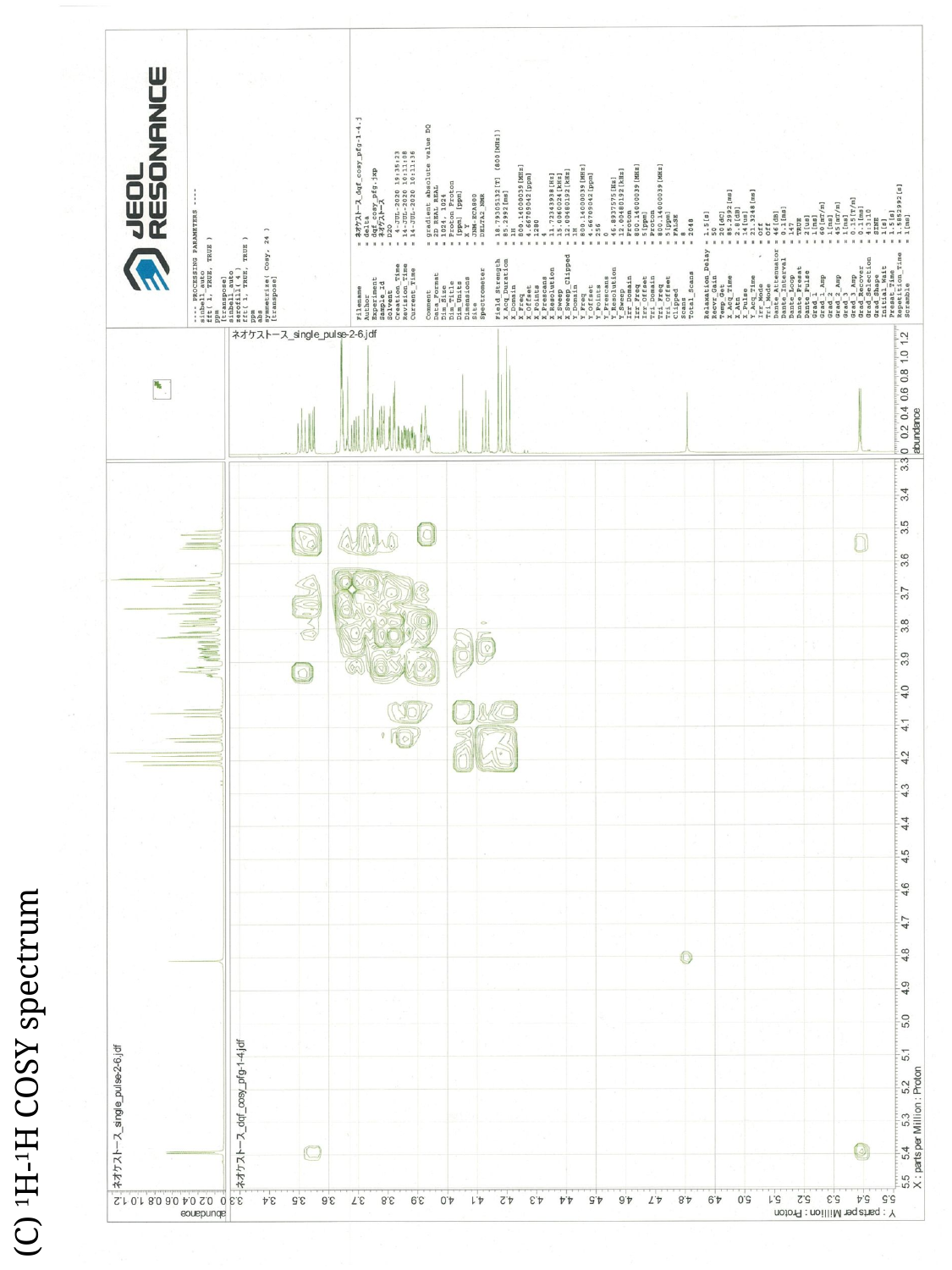

(C) 1H-1H COSY spectrum

## Slide 7
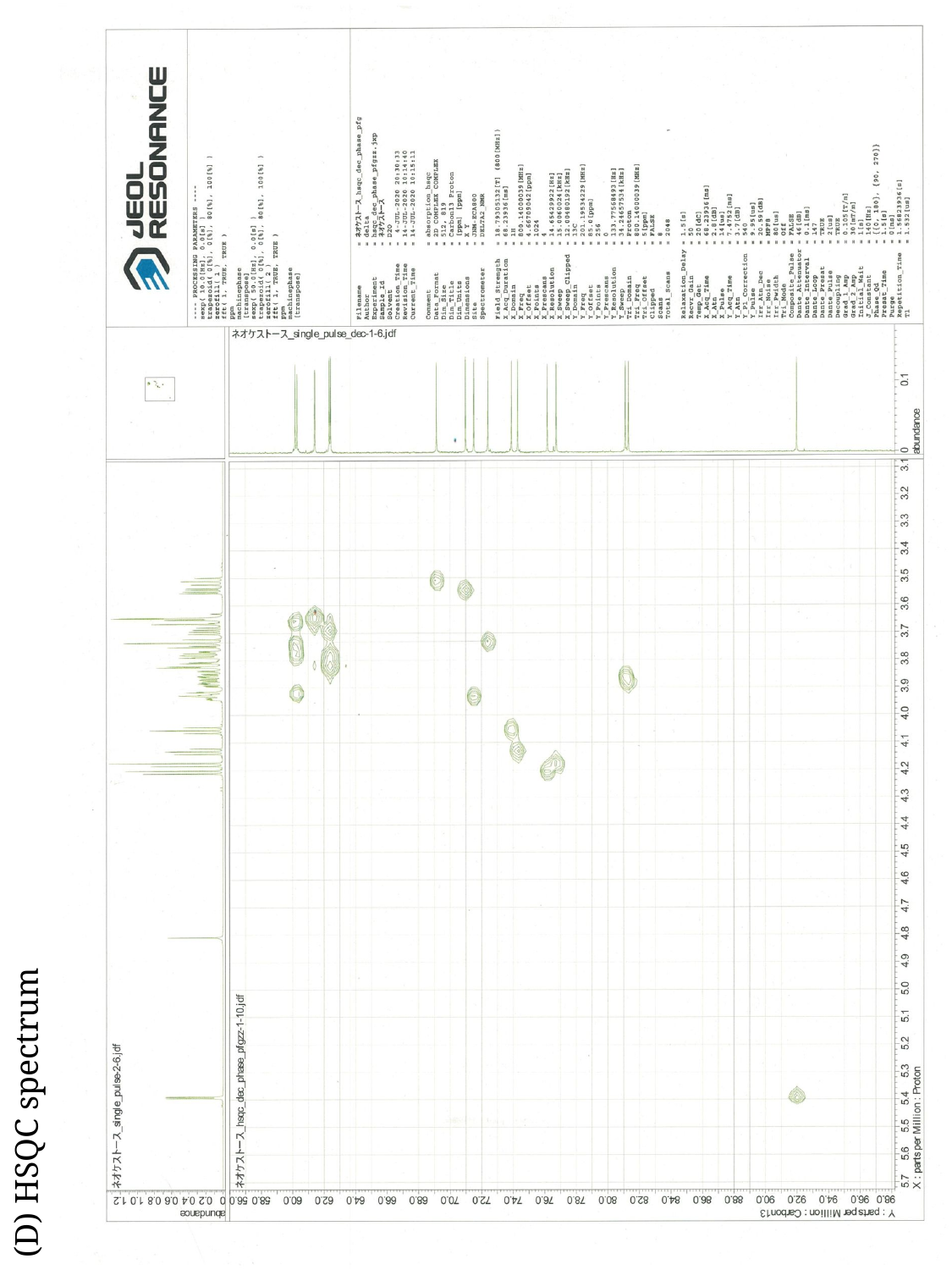

(D) HSQC spectrum

## Slide 8
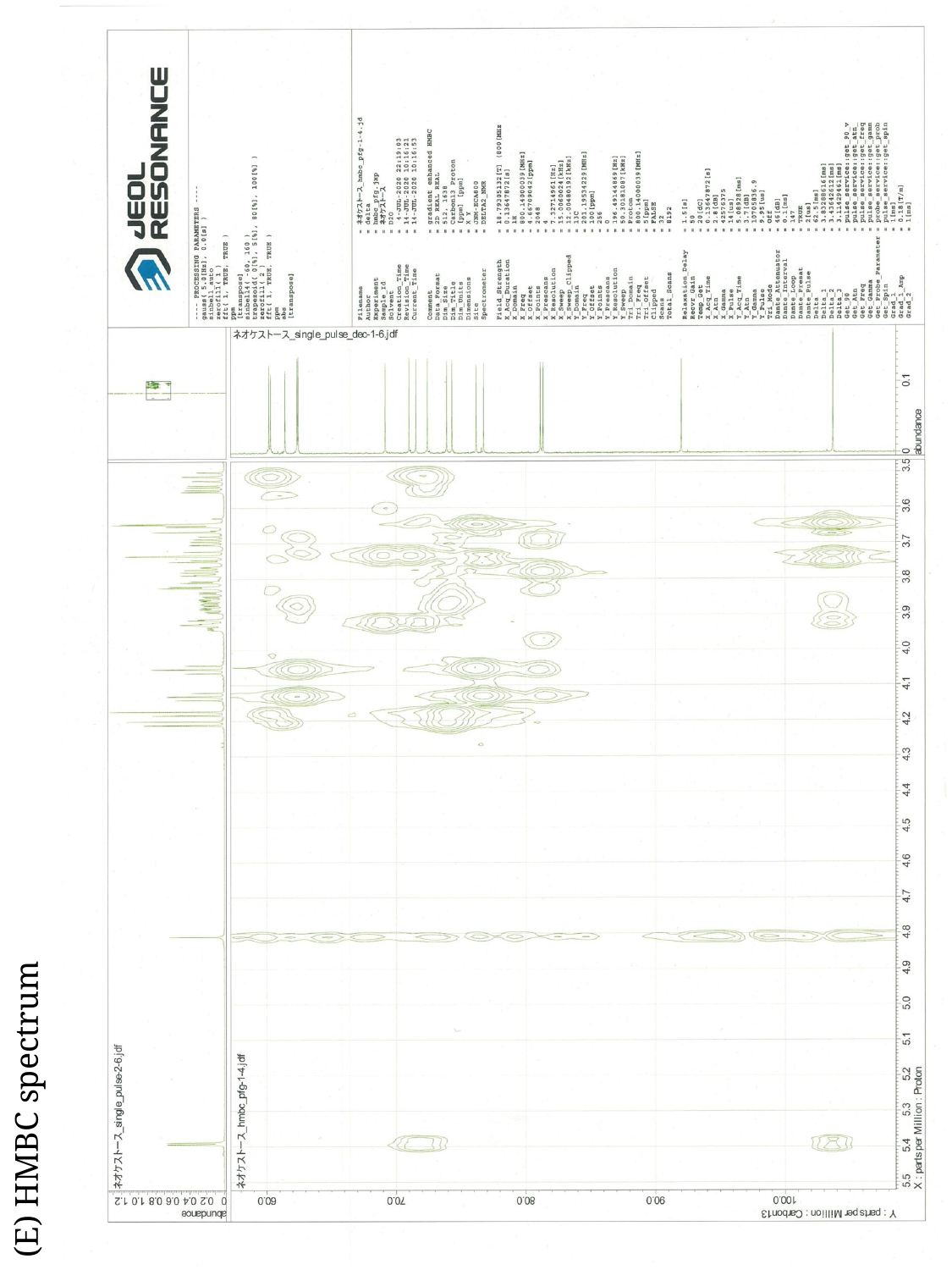

(E) HMBC spectrum

## Slide 9
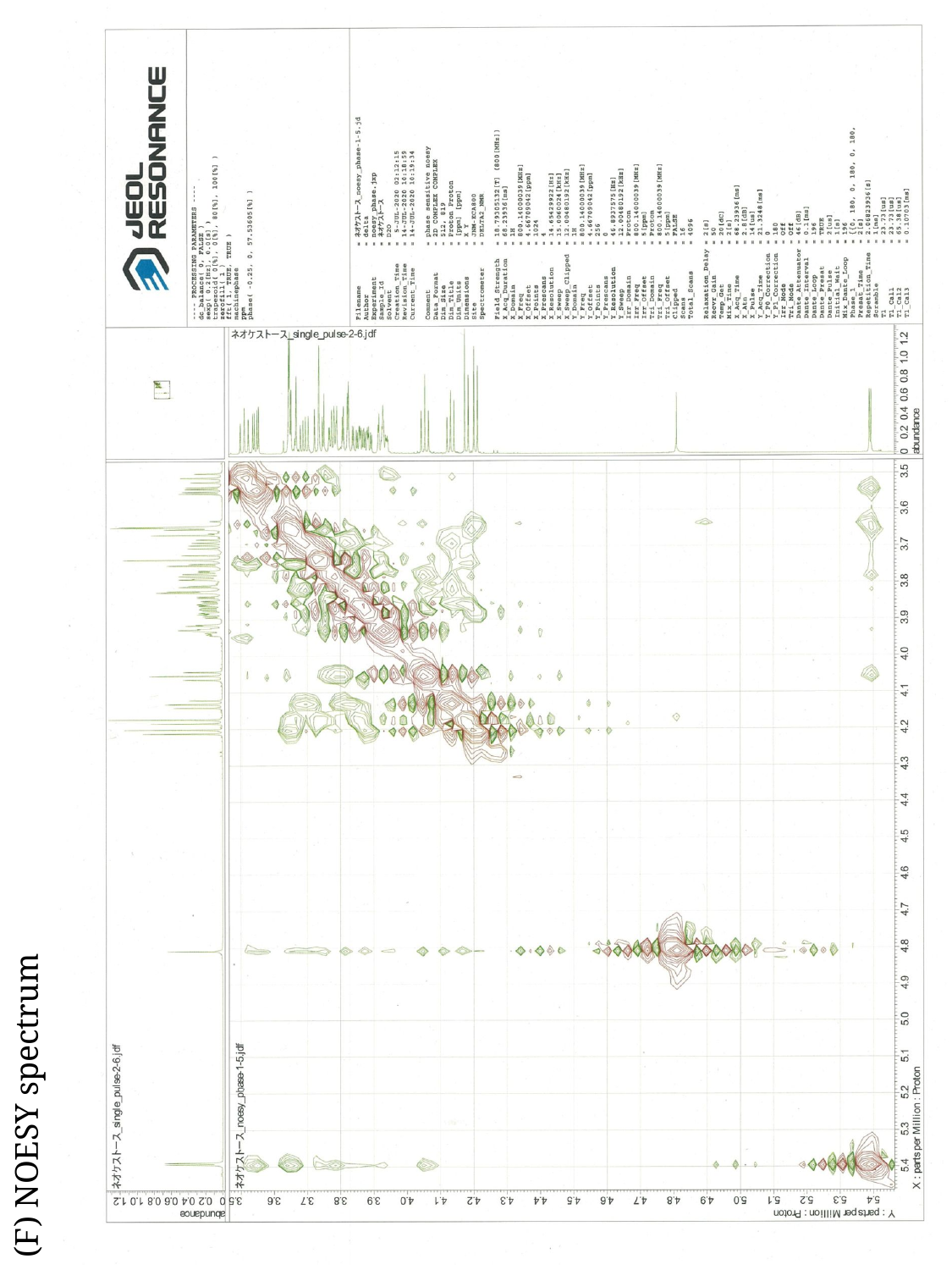

(F) NOESY spectrum

## Slide 10
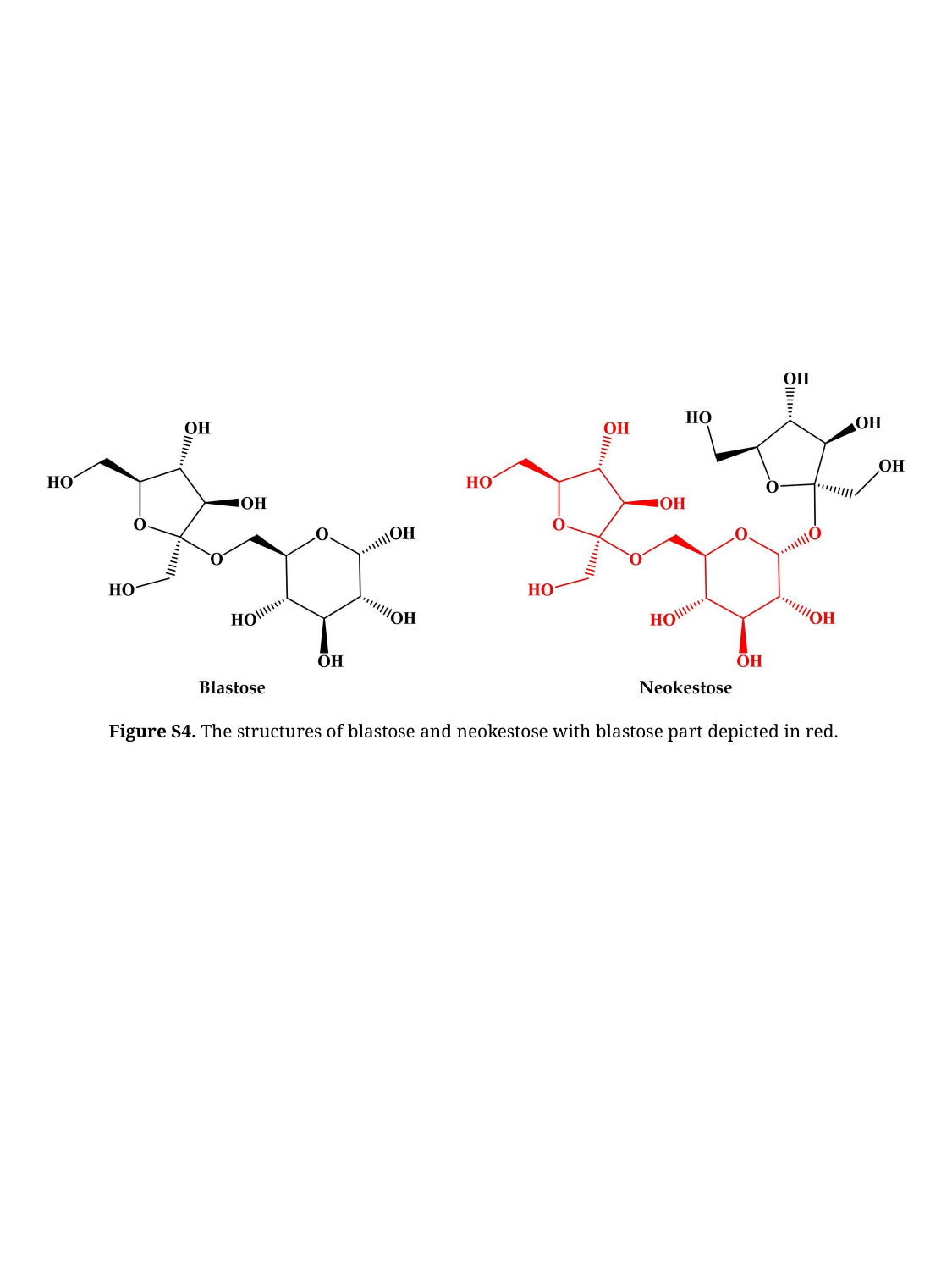

Figure S4. The structures of blastose and neokestose with blastose part depicted in red.
